# Manual Therapy Techniques Versus Occlusal Splint Therapy for Temporomandibular Disorders: A Systematic Review with Meta-Analysis

**DOI:** 10.3390/dj12110355

**Published:** 2024-11-01

**Authors:** Víctor Villar-Aragón-Berzosa, Esteban Obrero-Gaitán, Miguel Ángel Lérida-Ortega, María del Carmen López-Ruiz, Daniel Rodríguez-Almagro, Alexander Achalandabaso-Ochoa, Francisco Javier Molina-Ortega, Alfonso Javier Ibáñez-Vera

**Affiliations:** 1 Villar-Aragón Clinic, Calle Virgen de Guadalupe, 23400 Ubeda, Spain; vev00001@red.ujaen.es; 2 Department of Health Sciences, University of Jaen, Campus las Lagunillas s/n, 23071 Jaen, Spain; malerida@ujaen.es (M.Á.L.-O.); mlruiz@ujaen.es (M.d.C.L.-R.); aaochoa@ujaen.es (A.A.-O.); ajibanez@ujaen.es (A.J.I.-V.); 3 Hospital San Agustín, Andalusian Health Service, Avenida de San Cristóbal, 23700 Linares, Spain; 4 Department of Nursing, Physiotherapy and Medicine, University of Almeria, 04120 Almería, Spain; dra243@ual.es; 5 Department of Physiotherapy, University of Granada, Avenida de la Ilustración, 60, 18071 Granada, Spain; fjmolina@ugr.es; 6 Instituto de Investigación Biosanitaria ibs.Granada, 18012 Granada, Spain

**Keywords:** temporomandibular disorders, manual therapy, occlusal splints, pain, disability

## Abstract

**Background:** Manual therapy (MT) and occlusal splint therapy (OST) are the most conservative therapies applied on patients with temporomandibular disorders (TMDs). The aim was to compare the efficacy of MT vs. OST in improving pain, maximal mouth opening (MMO), disability, and health related-quality of life (hr-QoL) in these patients. **Methods:** According to PRISMA guidelines, a meta-analysis (CRD42022343915) was conducted including randomized controlled trials comparing the effectiveness of MT vs. OST in TMD patients, after searching in PubMed, PEDro, SCOPUS, and WOS up to March 2024. Methodological quality and risk of bias were assessed using the PEDro Scale. Cohen’s standardized mean difference (SMD) and its 95% confidence interval (95% CI) were the pooled effect measures calculated. **Results:** Nine studies, providing data from 426 patients, were included. Meta-analyses revealed that MT is more effective than OST in reducing disability (SMD = −0.81; 95% CI −1.1 to −0.54) and increasing MMO (SMD = 0.52; 95% CI 0.27 to 0.76) without differences for improving pain intensity and hr-QoL. Subgroup analyses revealed the major efficacy of OST in reducing pain in myogenic patients (SMD = 0.65; 95% CI 0.02 to 1.28). **Conclusions:** With caution, due to the low number of studies included, MT may be more effective than OST for improving disability and MMO in patients with TMDs.

## 1. Introduction

Temporomandibular disorders (TMDs) are the leading cause of non-dental pain in the orofacial region, including the head, face, and related structures [1]. TMDs are considered the second most common musculoskeletal condition that causes pain and disability worldwide [2,3] with an incidence of 34% in the worldwide population [4]. More specifically, TMDs affect approximately 29% of the European population (18% of children and 41% of adults) [4]. The main clinical sign and symptoms are difficulty or limitation in opening the mouth and pain in the temporomandibular joint (TMJ) [5,6], negatively affecting the health-related quality of life (hr-QoL) [7]. TMDs are often associated with other chronic pain conditions such as fibromyalgia or migraine, suggesting the involvement of the central sensitization mechanism in the development of TMDs [8]. TMDs are mainly classified following the Axis I of the Diagnostic Criteria for Temporomandibular Disorders (DC/TMD) in muscle disorders (Group I), disk displacement disorders (Group II), and Arthralgia/Arthritis/Arthrosis (Group III).

TMDs have been established as a public health problem requiring treatment. A great number of treatments have been suggested to manage this disorder: manual therapy (MT) [9], occlusal splint therapy (OST) [10], laser therapy [11], intramuscular injection of local anesthetic or botulinum toxin-a [12], intra-articular injections with hyaluronic acid [13], muscle relaxants, or oxidative ozone therapy [14].

Today, one of the most applied treatments for TMDs is OST, reducing the frequency and intensity of pain and increasing the maximum mouth opening (MMO) in these patients [10]. Full-coverage occlusal splints (OSs), or the Michigan type, are the most commonly used, usually being fabricated in a dental laboratory using methacrylate acrylic [15]. It has been observed that this type of splint guarantees a solid occlusal contact of the posterior teeth, absorbing the force of the muscular contraction and allowing a greater biting force but a lower joint load [15]. Thus, the Michigan OS would provide short-term pain relief for TMD patients [15]. Anterior repositioning appliances (ARAs) are another type of OSs that are also commonly used, usually in patients with TMDs or disk disorders [15]. These OSs are removable acrylic devices that are placed in the upper jaw and are designed to force the lower jaw to remain in a protruding position, and they are primarily indicated on a temporary basis for certain painful and inflammatory TMJ processes, as placing the lower jaw in an anterior position reduces the load on the joint, facilitates the healing process, and relieves joint pain during the healing process [15].

MT has been considered as one of the most effective approaches in the management of TMDs [16]. Due to the multifactorial etiology of the pathology and the complexity of TMDs, several approaches have been used, such as TMJ mobilization [17], cervical spine manipulation and mobilization [18,19], soft tissue techniques, and massage of the masticatory and cervical muscles [18,20,21]. Furthermore, the efficacy of MT in reducing pain and improving MMO and pressure pain threshold in subjects with TMD signs and symptoms has been previously reported [22].

Several systematic reviews have assessed the individual effects of different therapies in temporomandibular disorders: MT [22], MT added to therapeutic exercise [23], therapeutic exercise alone [24], or OST [25]. Moreover, a systematic review performed by Zhang et al. (2021) compared the effects of therapeutic exercise versus OST [26]. All these reviews show controversial results for MMO, pain, and quality of life, with a moderate to low quality of evidence. However, to our knowledge, no specific systematic review or meta-analysis comparing the efficacy of MT versus OST in the management of TMD patients has been carry out. Therefore, the aim of this study was to identify and retrieve all previously published evidence to clarify if MT is more effective than OST for reducing pain intensity and disability (severity of symptoms) and increasing MMO and hr-QoL in patients with TMDs.

## 2. Materials and Methods

A systematic review with meta-analysis was performed according to the Preferred Reporting Items for Systematic Reviews and Meta-Analyses (PRISMA) statement [27] and the recommendations of the Cochrane Handbook for Systematic Reviews of Interventions [28]. This review was registered in PROSPERO (CRD42022343915).

### 2.1. Literature Search

Two authors (V.V.-A.-B. and A.J.I.-V.), independently conducted a bibliographic search in the PubMed Medline, PEDro, Scopus, and Web of Science databases from inception up to March, 2024. In addition, the authors examined reference lists from retrieved full-length articles, previous published reviews, practice guidelines, expert documents, and the gray literature. The search tool proposed by the Cochrane Library based on the Population, Intervention, Comparison, Outcomes, and Study Design (PICOS) tool [28] was used to identify potential studies in the search: population (patients with TMDs), intervention (MT), comparison (OST), outcomes (pain intensity, disability, MMO, and hr-QoL), and study design (randomized controlled trials (RCTs) or RCT pilot). In accordance with the Medical Subject Headings from Medline, the keywords used in the search strategy were TMJ disorders, joint disorders, MT, musculoskeletal manipulations, osteopathic manipulation, or occlusal splints. According to each database, a specific keyword combination was used with the appropriate tags and Boolean ‘and’/‘or’ operators. We took as reference the search strategy designed for PubMed: (temporomandibular joint disorder*[mh] OR temporomandibular joint disease*[tiab] OR temporomandibular disease*[tiab] or “TMJ disorders”[tiab] or “TMJ diseases”[tiab]) AND (musculoskeletal manipulations[mh] OR musculoskeletal manipulation*[tiab] OR manipulation therap*[tiab] OR manual therap*[tiab]) AND (periodontal splint*[mh] OR occlusal splint[mh] OR dental night guard[tiab]) AND (pain[mh] or pain[tiab]). No year of publication or language filters were used. Doubts related to the inclusion of key terms, synonyms in the search strategy, and their application in the different databases were resolved by a third author with experience in the search strategy (E.O.-G.).

### 2.2. Study Selection

Two blinded reviewers (V.V.-A.B. and A.J.I.-V.) independently screened the titles and abstracts of all the references collected through the search strategy to identify potentially eligible studies to be included in the review. A third reviewer (F.J.M.-O.) solved any disagreements that arose during selection.

Only those studies meeting all the inclusion criteria were included in the review: (1) RCT or RCT pilot studies; (2) including participants with TMDs; (3) studies that compare, at least, two groups (MT and OST); and (4) studies with quantitative data of the outcomes of interest (mean or standard deviation post-therapy) to perform meta-analysis. Exclusion criteria included (1) RCT studies with intervention and comparison groups that were not exclusively composed of people with TMDs; (2) RCT studies that did not provide quantitative data susceptible to be transformed for inclusion in the quantitative synthesis of this review; and (3) non-randomized clinical trials.

### 2.3. Data Extraction

Two independent reviewers (M.Á.L.-O. and M.d.C.L.-R.), independently collected data from the included studies using a standardized data collection form designed by the authors in Microsoft Excel. Discrepancies were resolved by the participation of a third author (A.J.I.-V.). For each selected study, the characteristics collected were authorship and date of publication, study design, sample characteristics of the intervention, and comparison groups (sample size of each group, age, sex ratio, and TMD characteristics), interventions used in experimental and comparison groups (type of intervention, number of sessions, frequency of sessions, duration of therapy, or follow-up period) and outcomes (pain intensity, disability [severity of the symptoms], MMO, and hr-QoL).

### 2.4. Assessment of the Methodological Quality and of the Quality of Evidence

Two reviewers (D.R.-A. and A.A.-O.), independently judged the methodological quality of each study and the quality of the evidence of each finding. On the one hand, to assess methodological quality and risk of bias in the individual included studies, the PEDro Scale was used [29]. This tool is composed of 11 items that could be answered as “yes” (if item is met) or “no” (opposite). A score between 0 (high risk of bias) and 10 (null risk of bias) can be obtained, adding items 2 to 11 (item 1 is not included because it informs about external validity [30]). The methodological quality can be excellent (10–9 points), good (8–6 points), moderate (5–4 points), and low (3–0 points). Risk of bias in individual studies, inconsistency, indirectness, imprecision, and risk of publication bias were assessed using the GRADE system [31]. All these items, except the first, were evaluated according to the GRADE checklist by Meader et al. [32]. The overall quality of each meta-analysis was downgraded from high quality by one level for each factor we found. In the case of the presence of several limitations, the overall quality level was lowered by two levels. Finally, the level of evidence in each meta-analysis was classified as follows: (1) high (the findings are strong); (2) moderate (it is possible that further research could change our results); (3) low (the level of confidence in our set and the effect is very small); or (4) very low (any estimate of effect is very uncertain).

### 2.5. Statistical Analysis

*Comprehensive Meta-Analysis* (Biostat, Englewood, NJ, USA) was the statistical software employed to perform the meta-analysis by the two authors (E.O.-G. and A.J.I.-V.). Based on the recommendations of Cooper et al. (2009) [33] and with the aim to generalize our findings taking into account heterogeneity between studies, we used a fixed- or random-effects model to estimate the pooled effect [34]. Effect size was calculated using Cohen’s standardized mean difference (SMD) and its 95% confidence interval (95% CI) [35], and could be null (SMD 0), low (SMD 0.2), medium (SMD 0.5), or large (SMD > 0.8). Findings in meta-analyses were graphically displayed in the forest plots [36]. It is recommended that the risk of publication bias is assessed using more than one method, especially if the studies included are 10 or less [37]. Due to this, the risk of publication bias was assessed with data from at least two or more studies, and it was studied visualizing the funnel plot [38] and *p*-value for the Egger test [39]. The agreement between these tests was poor, and, additionally, we estimated the risk of publication bias applying the trim-and-fill method [40]. If funnel plot was asymmetric, *p* for Egger test < 0.1, and variation between the adjusted and original SMD in the trim-and-fill estimation was >10%, it indicated the presence of publication bias risk. Related to publication bias, the quality level of evidence was downgraded if the adjusted SMD varied more than 10% with respect to the original SMD [41]. To calculate statistical heterogeneity, we used the *p*-value for the Q-test (*p* < 0.1 indicates heterogeneity) and the degree of inconsistency (I^2^) from Higgins et al. that categorizes heterogeneity as null (0%), low (<25%), moderate (25–50%), and large (>50%) [42,43].

As additional analyses sensitivity and subgroup analyses were carried out, the sensitivity analysis was performed by the exclusion method or the leave-one-out method, which consisted of performing a meta-analysis of each subset of the studies obtained and omitting one study to show how each individual study affects the global estimate of the rest of the studies. By this, the contribution of each study to the pooled effect in each meta-analysis was determined [28]. The subgroup analysis, when possible (*k* > 2 per subgroup), compared the effectiveness of both therapies according to the type of TMD patients: arthrogenic or myogenic TMDs.

## 3. Results

### 3.1. Study Selection

**Figure 1:**
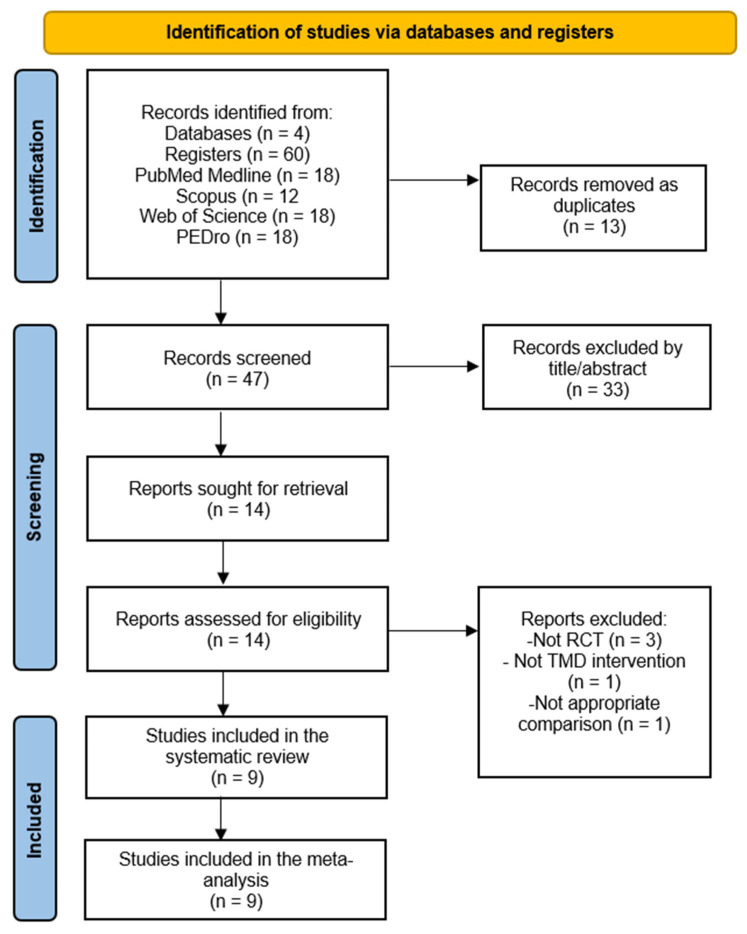
PRISMA flow chart.

Sixty records were retrieved through the databases. After excluding duplicates (*n* = 13), 47 records were screened by title and abstract, excluding 33 for this reason and 5 for not meeting the inclusion criteria (Supplementary File S1). Finally, nine RCTs were included in this systematic review with a meta-analysis [20,44,45,46,47,48,49,50,51]. Figure 1 shows the PRISMA flow diagram.

### 3.2. Characteristics of the Studies Included in the Review

The studies included were carried out between 2010 and 2019 in countries such as Brazil, the United States, Italy, Germany, The Netherlands, and Japan. A total of 426 patients with TMDs were provided by the studies included with a mean age of 33.3 ± 4.2 years old (64% female). To establish the TMD diagnosis, five studies using the RDC/TMD diagnostic criteria [45,47,48,49,51], and in four studies the diagnosis was made using the Temporomandibular Index (TMI) [44], the Fonseca Index [20,46], and a magnetic resonance image [50]. One hundred and eighty patients received MT (including physiotherapy programs-based massage, osteopathic therapy, exercises, and mobilizations) and 246 patients received OST. All studies included reported data from the immediate effect of MT and OST. Related to variables were the pain was assessed with data from the visual analog scale and numeric pain rating scale; MMO, with data from assessments using calibrated calipers; disability, with data from the Fonseca Patient History Index, the ProTMDMulti assessment and the TMD Questionnaire; and hr-QoL with data from the Oral Health Impact Profile-14. Only three studies received external funding. Table 1 shows the main characteristics of the studies included.

### 3.3. Assessment of Methodological Quality and Main Biases Identified

Table 2 reports the PEDro score for each study included in the review. The studies included showed moderate methodological quality and medium risk of bias (PEDro score 5.6 ± 1.1 points). Four studies showed good methodological quality and five showed moderate quality. In any study, participants and therapists were blinded, increasing the risk of performance biases. Detection bias, due to evaluators who were not blinded, was present in four studies.

### 3.4. Main Findings in Meta-Analyses

#### 3.4.1. Pain

**Figure 2:**
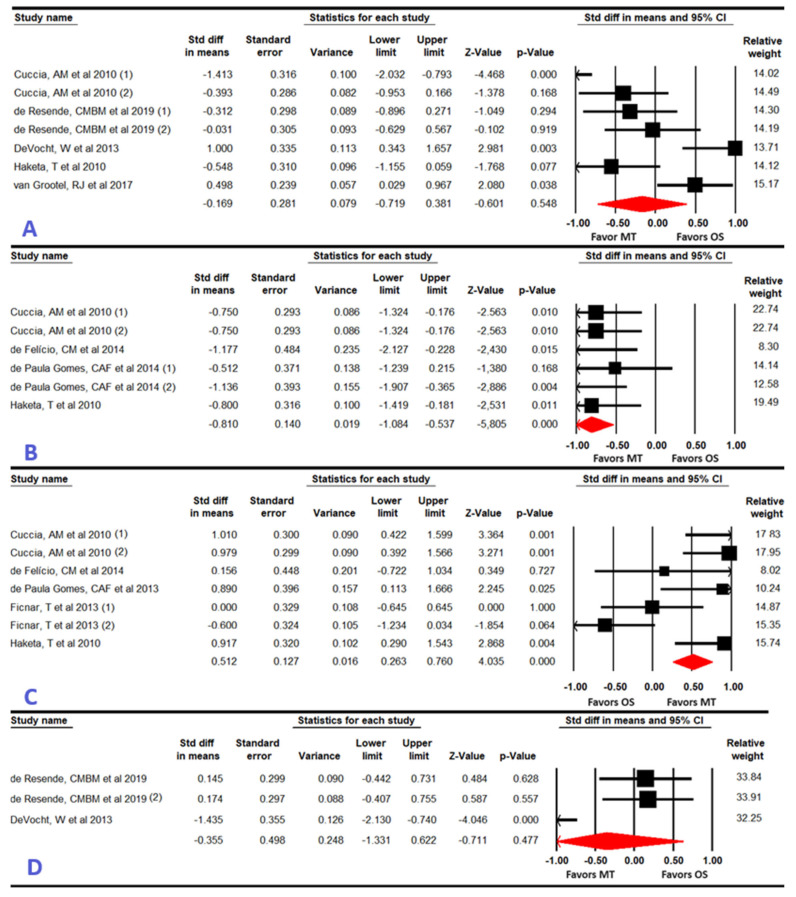
Forest plot for pain intensity (**A**) [44,47,48,50,51], disability (**B**) [44,45,46,50], maximal mouth opening (**C**) [20,44,45,49,50], and health related-quality of life (**D**) [47,48].

Five studies with seven independent comparisons [44,47,48,50,51] provided data to assess the effect of MT, compared to OSs, in reducing pain intensity. Our findings did not reveal statistically significant differences between therapies (SMD = −0.17; 95% CI −0.72 to 0.38; *p* = 0.55) (Table 3, Figure 2). Although risk of publication bias was confirmed (asymmetric funnel plot and *p* for Egger = 0.07), the adjusted SMD using the trim-and-fill estimation was not statistically significant (SMD = 0.03; 95% CI −0.57 to 0.67), so the original findings did not change (Supplementary Figure S1). Heterogeneity was low (I^2^ = 6.4%, Q = 6.4; df = 6; *p* = 0.38). The sensitivity analysis did not show differences.

Subgroup analysis revealed that OST is more effective than MT in reducing pain only in patients with myogenic TMDs just to the finish the intervention (SMD = 0.65; 95% CI 0.02 to 1.28; *p* = 0.04).

#### 3.4.2. Disability (Severity of the Symptoms)

This meta-analysis was performed with data from four studies with six independent comparisons [44,45,46,50]. Our findings showed that a large effect (SMD = −0.81; 95% CI −1.1 to −0.54; *p* < 0.001) favors MT, with respect to OSs, in reducing disability (Table 3, Figure 2) without risk of publication bias or heterogeneity (I^2^ = 0%, Q = 2; df = 5; *p* = 0.32). No differences were found in the sensitivity analysis.

#### 3.4.3. Maximal Mouth Opening

Five studies with seven independent comparisons [20,44,45,49,50] provided data to assess the efficacy of MT vs. OSs in improving MMO ROM. Our meta-analysis showed a medium effect (SMD = 0.52; 95% CI 0.27 to 0.76; *p* < 0.001) that favors MT (Table 3, Figure 2). No risk of publication bias was found and heterogeneity was null (I^2^ = 0%, Q = 5.6; df = 6; *p* = 0.47). No differences were found in the sensitivity analysis.

#### 3.4.4. Health Related-Quality of Life

This meta-analysis was performed with data from two studies with three independent comparisons [47,48]. No statistically significant differences (SMD = −0.36; 95% CI −1.33 TO 0.62; *p* = 0.477) were found between MT and OSs to improve hr-QoL (Table 3, Figure 2). No risk of publication bias was present and heterogeneity was low (I2 = 10.5%, Q = 2.3; df = 2; *p* = 0.32). The sensitivity analysis did not show differences.

## 4. Discussion

TMDs are common conditions in the general population characterized by pain and dysfunction around the TMJ area. The prevalence of TMDs has increased in recent years, thus the design of an effective treatment for these conditions is a key objective in research in this field, thereby reducing associated healthcare costs [52]. Several treatments have been proposed for the management of TMDs, including OST, MT, and drug treatment. To the best of our knowledge, this is the first specific systematic review with meta-analysis focused on the comparison of the effectiveness of two of the main frequent treatments for TMDs, OST and MT. In the present meta-analysis, we have analyzed nine studies with a total sample size of 426 patients. The results of our analysis demonstrate that both occlusal splint therapy (OST) and manual therapy (MT) are effective in reducing pain and improving the quality of life in patients with temporomandibular joint (TMJ) disorders. Nevertheless, the findings indicate that MT may be more efficacious than OST in improving TMJ function, as evidenced by an increase in MMO and a reduction in disability.

In clinical practice, pain is the primary symptom observed in patients with TMDs. This pain is primarily of myogenic origin and appears to be associated with jaw dysfunction [53]. It is noteworthy that the majority of patients included in this meta-analysis appear to experience myogenic pain, either exclusively or in conjunction with other types of pain. Previous research has indicated that MT may offer pain relief for TMD patients when compared with a control group [54]. Conversely, a recent systematic review has indicated that the OST may be an ineffective intervention for reducing pain in subjects with myogenic TMDs when compared with a sham group [55]. The results of our meta-analysis contrast with these previous results. That is, we have not detected a better pain management for MT when compared to OST, probably because we have carried out a quantitative analysis of the data and not only a qualitative analysis. On the contrary, the present work shows similar results to those that revealed no differences for pain reduction in TMD patients when comparing exercise vs. OST [56] or LASER vs. OST [57]. Taking all these results together, it can be suggested that physical therapy treatments, including MT, have similar effects on pain reduction that OST has on patients with TMDs. Furthermore, the subgroup analysis of the subsets of participants indicated that OST treatment is superior to MT in improving temporomandibular pain in patients with myogenic TMDs. In contrast with this result, a previous meta-analysis indicated that MT is more effective than OST for pain relief in patients with myogenous temporomandibular disorders in the short-term (<5 months) [16]. Nevertheless, our data should be taken into account in future studies, as our work represents the first to specifically compare MT versus OST. However, it is important to acknowledge the limited quality of the evidence of this result.

On the other hand, our findings indicate that MT has a superior effect on maximum mouth opening (MMO) and temporomandibular function (decreasing disability) compared with OST in TMD patients. This result may be explained considering that the main tissues affected by MT techniques are the connective and the skeletal muscle tissue. Thus, the application of massage, passive jaw mobilization, or stretching techniques may serve to reduce connective tissue and skeletal muscle stiffness, thereby facilitating a more pronounced increase in functional capacity [58,59]. Even so, this is an intriguing result, given that previous studies have demonstrated a positive correlation between pain and temporomandibular function [53,60,61]. In this line, we have found an improvement in temporomandibular function in the MT group compared with the OST group, with a high effect size, without finding significant differences in pain between the MT and OST groups. This result could be due to two reasons. Firstly, the studies included in our meta-analysis collected the pain data using subjective methods (e.g., the numerical rating scale or visual analogic scale), while the maximum mouth opening value was obtained using an objective method (e.g., distance between upper and lower incisors with calipers). Secondly, as evidenced in the scientific literature, the experience of pain is influenced by a number of subjective factors such as previous experiences, fear-avoidance behaviors, and beliefs, among other [62,63]. Therefore, it can be considered that pain is a subjective output of the central and peripheral nervous systems, which may manifest or be intensified in a multitude of syndromes or pathologies, without the existence of real or proportional tissue damage or dysfunction.

The present study also analyzed the efficacy of occlusal splint therapy (OST) and myofascial release (MT) in reducing the severity of TMD symptoms. Our results indicate that MT is superior to OMT to diminish the severity of the symptoms perceived by TMD patients. This is also an intriguing outcome given that, as previously stated, MT does not appear to be more effective than OST in reducing pain in TMD patients. Patients with TMDs present a range of symptoms affecting the jaw and adjacent areas, including pain, muscle tenderness, clicking noises, and limited range of motion of the temporomandibular joint [6]. The studies included in our meta-analysis evaluate the severity of the symptoms using questionnaires that analyze all of these symptoms. For this reason, our statistical analysis suggests that MT is better than OST for reducing the global symptomatology in TMD patients.

Finally, our meta-analysis did not show superiority to any treatment to improve the quality of life in TMD patients. This result may be attributed to the significant impact that temporomandibular pain has on the quality of life of these patients [64,65]. Given that our findings indicate comparable efficacy of both treatments (OST and MT) in alleviating pain in TMD patients, no notable enhancement in quality of life was detected when MT and OST were contrasted in our meta-analysis. An additional probable explanation for this outcome is the limited number of comparisons included in our meta-analysis that examine this variable.

Although the findings present in this work present clinical relevance, it is important to consider some limitations. The inclusion of a limited number of studies and the relatively small sample sizes in some of these studies may reduce the robustness, quality of evidence, and generalizability of our findings. Another limitation is the potential for publication bias to influence several of our results. Additionally, it is important to note that the possibility of patient follow-up could not be considered for the studies included due to the limitations of their design. Future studies with larger sample sizes are necessary to elucidate the impact of MT versus OST on pain, function, and quality of life in TMD patients.

## 5. Conclusions

The findings of this systematic review with a meta-analysis indicate that MT may be a more effective intervention than OST for improving MMO and reducing the severity of disabling symptoms in patients with TMDs. On the contrary, there is a possibility that MT may not be more effective than OST in alleviating pain and consequently improving HR-QoL in this patient group. Only for patients with myogenic TMDs is OST suggested to be superior to MT, but with a very low level of evidence and caution in the generalization. Furthermore, OST seems to be superior to MT in the management of myogenic TMD pain. In view of our results, OST in combination with MT should be applied to the treatment protocols for TMDs. However, further studies are required to enhance the generalizability, evidence base, and robustness of these findings.

## Figures and Tables

**Table 1:** Characteristics of the studies included.

		MT Group		OST Group		Outcomes	
Study	Number of Patients	Sample Characteristics	Intervention Characteristics	Sample Characteristics	Intervention Characteristics	Variable	Test
Cuccia et al. 2010 (Italy) [44] Design: RCT Setting: Department of Orthodontics and Gnathology, University of Palermo, Italy Funding: No	50 patients diagnosed with TMDs (18–50 years old; 28F:22M)	25 patients (40.6 years old; 13F:12M)	Osteopathic manipulation directed to the cervical and TMJ regions. Treatments lasted 15–25 min and were gentle techniques such as myofascial release, balanced membranous tension, muscle energy, myofascial release, joint articulation, high velocity-low-amplitude thrust, and cranial-sacral therapy.	25 patients (38.4 years old; 15F:10M)	Oral appliance therapy, physical therapy (gentle muscle stretching and relaxing exercises), therapies such as hot or cold packs (or both), and transcutaneous electrical nerve stimulation.	Pain intensity	VAS
						MMO	Calibrated caliper in millimeters
						Disability	Temporomandibular Index
Haketa et al. 2010 (Japan) [50] Design: RCT Setting: Clinic of the Tokyo Medical and Dental University Funding: No	44 patients with TMDs (38.7 years old; 40F:4M)	19 patients (38.8 years old; 19F:0M)	Manual therapy based-self-care passive protocol and exercises	25 patients (38.6 years old; 21F:4M)	Stabilization splint therapy	Pain	VAS
						MMO	Calibrated caliper in millimeters
Ficnar et al. 2013 (Germany) [49] Design: RCT Setting: Department of Prosthetic Dentistry and Biomaterials and the Department of Orthodontics of the Center for Dental, Oral and Maxillofacial Diseases of Münster University Hospital Funding: Yes	58 patients with TMDs (median age of 34.6 years old; 50F:8M)	19 patients	Manual therapy (including massage techniques and exercises)	18 patients	Occlusal appliance therapy every night and two hours during the day	MMO	Calibrated caliper in millimeters
				21 patients	Occlusal appliance therapy every night and two hours during the day		
DeVocht et al. 2013 (United States) [48] Design: RCT pilot Setting: University of Iowa Funding: No	40 TMD patients diagnosed (mean of 35 years old; 33F:7M)	20 patients (31.7 years old; 16F:4M)	12 sessions along 2 months of chiropractic techniques applied to all biomechanical jaw dysfunctions	20 patients (36.9 years old; 17F:3M)	Reversible inter-occlusal splint therapy	Pain intensity	Numeric Pain Rating Scale
						Health related-Quality of Life	Oral Health Impact Profile-14
de Felicio et al. 2014 (Brazil) [45] Design: RCT Setting: Not reported Funding: No	20 patients with TMDs (13–64 years old; sex data not reported)	10 patients (31 years old)	Orofacial myofunctional therapy (massage, mobility, strength and coordination exercises, etc.) for 120 days (45 min each session)	10 patients (29 years old)	Occlusal splint therapy according to the Michigan principles	MMO	Calibrated caliper in millimeters
						Disability	ProTMDMulti
de Paula Gomes et al. 2014a (Brazil) [20] Design: RCT Setting: University of Sao Paulo, Brazil Funding: No	28 patients with TMDs (18–40 years old; 20F:8M)	14 patients (30.1 years old; 10F:4M)	Massage therapy involving sliding and kneading maneuvers on the masseter and temporal muscles. It was applied in sessions of 30 min, 3 times per week for 4 weeks.	14 patients (29.7 years old; 10F:4M)	Occlusal splint therapy for 4 weeks	MMO	Calibrated caliper in millimeters
de Paula Gomes et al. 2014b (Brazil) [46] Design: RCT Setting: University of Sao Paulo, Brazil Funding: No	59 patients with TMDs (18–40 years old; 50F:10M)	15 patients (29.3 years old; 13F:2M)	Massage therapy for 30 min using maneuvers of sliding and kneading on masseter and anterior temporal muscles, bilaterally). Twelve sessions for 4 weeks.	15 patients (27.8 years old; 12F:3M)	Conventional occlusal splint therapy	Disability	Fonseca Patient History Index
				14 patients (28.9 years old; 10F:4M)	Silicone (3 mm soft polyvinyl sheet) occlusal splint therapy		
Van Grootel et al. 2017 (The Netherlands) [51] Design: RCT Setting: Department in Utrecht and community individuals Funding: Yes	72 patients with myogenous TMDs (67F:5M)	37 patients (31.4 years old; 35F:2M)	Physiotherapy program (self-massage and exercises)	35 patients (29 years old; 32F:3M)	Occlusal splint therapy	Pain	VAS
de Resende et al. 2019 (Brazil) [47] Design: RCT Setting: Integrated Center for Care of Stomatognathic Dysfunction, Rio, Brazil Funding: Yes	70 patients with TMDs (18–60 years old)	21 patients	Manual therapy in sessions lasting 40 min, twice per week for 4 weeks.	24 patients	Occlusal splint therapy	Pain intensity	VAS
				25 patients	Occlusal splint therapy and more counseling	Health related-Quality of Life	Oral Health Impact Profile-14

Abbreviations: MT, manual therapy; OST, occlusal splint therapy; RCT, randomized controlled trial; F, female; M, male; TMJ, temporomandibular joint; VAS, visual analog scale; MMO, maximum mouth opening; TMDs, temporomandibular disorders.

**Table 2:** Methodological quality assessment (PEDro score).

Study	I1	I2	I3	I4	I5	I6	I7	I8	I9	I10	I11	Total	Quality
Cuccia et al. 2010 * [44]	Y	Y	N	Y	N	N	Y	Y	N	Y	Y	6/10	Good
Haketa et al. 2010 * [50]	Y	Y	Y	Y	N	N	N	N	N	Y	Y	5/10	Moderate
Ficnar et al. 2013 * [49]	Y	Y	N	Y	N	N	N	Y	N	Y	Y	5/10	Moderate
de Vocht et al. 2013 * [48]	Y	Y	Y	Y	N	N	N	N	Y	Y	Y	6/10	Good
de Felicio et al. 2014 * [45]	Y	Y	N	Y	N	N	N	N	N	Y	Y	4/10	Moderate
de Paula Gomes et al. 2014a [20]	Y	Y	Y	Y	N	N	Y	Y	N	Y	Y	7/10	Good
de Paula Gomes et al. 2014b [46]	Y	Y	Y	N	N	N	Y	Y	Y	Y	Y	7/10	Good
van Grootel et al. 2017 * [51]	N	Y	N	Y	N	N	Y	N	N	Y	Y	5/10	Moderate
de Resende et al. 2019 [47]	Y	Y	Y	N	N	N	Y	N	N	Y	Y	5/10	Moderate

Abbreviations: I1, eligibility criteria; I2, randomized distribution; I3, allocation concealment; I4, comparability at baseline; I5, blinded subjects; I6, blinded therapists; I7, blinded assessors; I8, adequate monitoring; I9, intention-to-treat analysis; I10, between-groups comparison; I11, point estimation and variability; Y, Yes; N, No. Note: Item 1 does not contribute to the final score. Note: * Score confirmed in PEDro webpage.

**Table 3:** Main findings in meta-analyses.

Outcomes	Summary of Findings									Quality Evidence (GRADE Assessment)					
	Effect Size				Heterogeneity		Publication Bias								
	K	SMD	95% CI	*p*	Q (df)	I^2^ (*p*)	Egger *p*	Trim-and-Fill		Risk Bias	Incon	Ind	Imp	Pub Bias	Quality
								Adj SMD	% var						
Pain intensity	7	−0.17	−0.72 to 0.38	0.548	6.4 (6)	6.4% (0.38)	0.26	0.03	100%	Medium	Low	No	Yes	Yes	Low
Disability	6	−0.81	−1.1 to −0.54	<0.001	2 (5)	0% (0.85)	0.71	−0.81	0%	Medium	No	No	No	No	Low
Maximal mouth opening	7	0.52	0.27 to 0.76	<0.001	5.6 (6)	0% (0.47)	0.65	0.52	0%	Medium	No	No	No	No	Low
Health related-quality of life	3	−0.36	−1.33 to 0.62	0.48	2.3 (2)	10.5% (0.32)	0.17	−0.36	0%	Medium	Low	No	No	No	Very low

Abbreviations: K, number of comparisons; SMD, Cohen’s standardized mean difference; 95% CI, 95% confidence interval; *p*, *p*-value; Q, Q-test; df, degree of freedom; I^2^, degree of inconsistency; Adj, adjusted; % var; percentage of variation; Incon, inconsistency; Ind, evidence indirect; Imp, imprecision; Pub, publication.

## Data Availability

Request to the corresponding author.

## Supplementary Materials

The following supporting information can be downloaded at: https://www.mdpi.com/article/10.3390/dj12110355/s1, Figure S1: Funnel plot for pain intensity. Supplementary File S1: Studies excluded with reasons.

## List of Abbreviations

TMDs: temporomandibular disorders; OST: occlusal splint therapy; MT: manual therapy; MMO: maximal mouth opening; hr-QoL: health-related quality of life.
