# Supplementary material for: Manual Therapy Techniques Versus Occlusal Splint Therapy for Temporomandibular Disorders: A Systematic Review with Meta-Analysis
Source: Dent J (Basel). 2024 Nov 1;12(11):355. doi: 10.3390/dj12110355 (PMC11593169; doi:10.3390/dj12110355)
Supplement: Supplementary file 1 [file dentistry-12-00355-s001.zip › Supplementary File S1. Studies excluded with reasons.pdf]

SUPPLEMENTARY FILE S1:
STUDIES THAT DID NOT MEET THE INCLUSION CRITERIA

Reason: Study design not accepted.

These 3 studies are not RCTs.

- Wieckiewicz M, Boening K, Wiland P, Shiau YY, Paradowska-Stolarz A. Reported concepts for the treatment modalities and pain management of temporomandibular disorders. J Headache Pain. 2015;16:106. doi: 10.1186/s10194-015-0586-5.
- Incorvati C, Romeo A, Fabrizi A, Defila L, Vanti C, Gatto MRA, Marchetti C, Pillastrini P. Effectiveness of physical therapy in addition to occlusal splint in myogenic temporomandibular disorders: protocol of a randomised controlled trial. BMJ Open. 2020 Aug 13;10(8):e038438. doi: 10.1136/bmjopen-2020-038438.
- Zhang L, Xu L, Wu D, Yu C, Fan S, Cai B. Effectiveness of exercise therapy versus occlusal splint therapy for the treatment of painful temporomandibular disorders: a systematic review and meta-analysis. Ann Palliat Med. 2021 Jun;10(6):6122-6132. doi: 10.21037/apm-21-451.

Reason: No statistical comparison between groups (1 study)

- Melo RA, de Resende CMBM, Rêgo CRF, Bispo ASL, Barbosa GAS, de Almeida EO. Conservative therapies to treat pain and anxiety associated with temporomandibular disorders: a randomized clinical trial. Int Dent J. 2020 Aug;70(4):245-253. doi: 10.1111/idj.12546.

Reason: Not accepted experimental intervention (TMD) carried out in this study:

- de Felício CM, de Oliveira MM, da Silva MA. Effects of orofacial myofunctional therapy on temporomandibular disorders. Cranio. 2010 Oct;28(4):249-59. doi: 10.1179/crn.2010.033.
