# Supplementary figures and images for: Manual Therapy Techniques Versus Occlusal Splint Therapy for Temporomandibular Disorders: A Systematic Review with Meta-Analysis
Source: Dent J (Basel). 2024 Nov 1;12(11):355. doi: 10.3390/dj12110355 (PMC11593169; doi:10.3390/dj12110355)

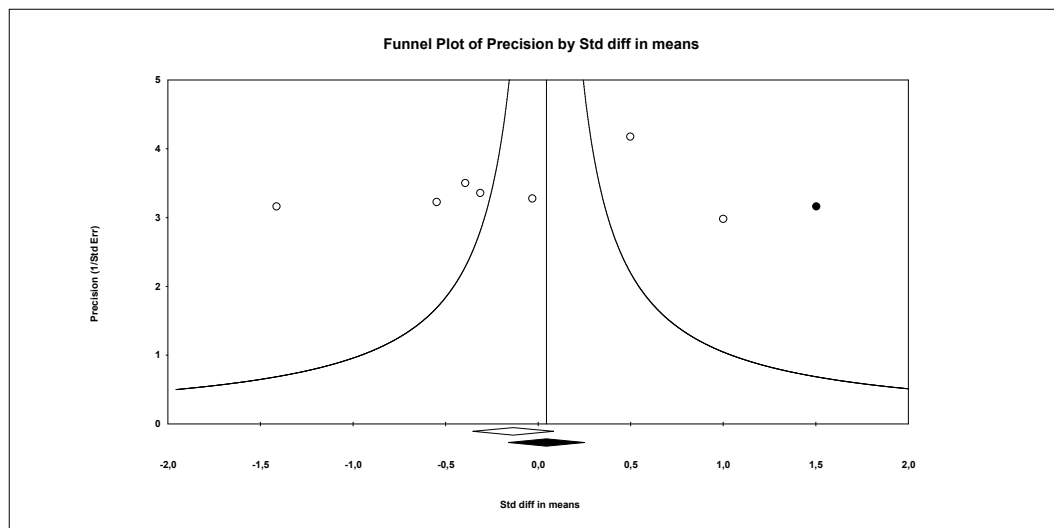

**Suppelementary Figure S1.** Funnel plot for pain intensity

Supplement: Supplementary file 1 [file dentistry-12-00355-s001.zip › Supplementary Figure S1.pdf]
